# Pharmacological update of mirtazapine: a narrative literature review

**DOI:** 10.1007/s00210-023-02818-6

**Published:** 2023-11-09

**Authors:** Emad H. M. Hassanein, Hanan S. Althagafy, Mohammad A. Baraka, Esraa K. Abd-alhameed, Islam M. Ibrahim

**Affiliations:** 1 Department of Pharmacology and Toxicology, Faculty of Pharmacy, Al-Azhar University, Assiut Branch, Assiut, 71524 Egypt; 2 Department of Biochemistry, Faculty of Science, University of Jeddah, Jeddah, Saudi Arabia; 3 Faculty of Pharmacy, Al-Azhar University, Assiut Branch, Assiut, 71524 Egypt; 4 Department of Pharmacology and Toxicology, Faculty of Pharmacy, Beni-Suef University, Beni-Suef, Egypt

**Keywords:** Mirtazapine, Antidepressant, CNS disorders

## Abstract

Mirtazapine (MTZ) is an antidepressant drug with an exceptional pharmacological profile. It also has an excellent safety and tolerability profile. The present review provides a pharmacological update on MTZ and summarizes the research findings of MTZ’s effects on different diseases. MTZ is hypothesized to have antidepressant effects because of the synergy between noradrenergic and serotonergic actions and is effective in treating major depressive disorder and depression associated with epilepsy, Alzheimer’s disease, stroke, cardiovascular disease, and respiratory disease. In cancer patients, MTZ significantly reduced sadness, nausea, sleep disruption, and pain and improved quality of life. Also, it has promising effects on Parkinson’s disease, schizophrenia, dysthymia, social anxiety disorder, alcohol dependency, posttraumatic stress disorder, panic disorder, pain syndromes, obsessive–compulsive disorder, and sleep disorders. Additionally, MTZ is potentially therapeutic in different situations associated with depression, such as liver, kidney, cardiovascular, respiratory, infertility, heavy metal-induced neurotoxicity, and pruritus. Potent antioxidative, anti-inflammatory, and anti-apoptotic bioactivities mediate these promising effects. These positive outcomes of the scientific investigations motivate more and more clinical trials for a golden exceptional antidepressant in different conditions.

**Graphical Abstract:**

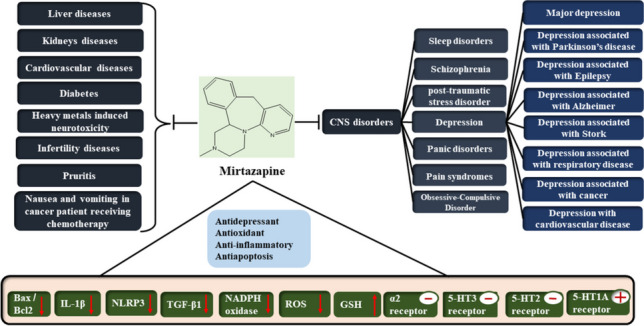

## Introduction

### Mirtazapine

#### Chemistry

**Fig. 1:**
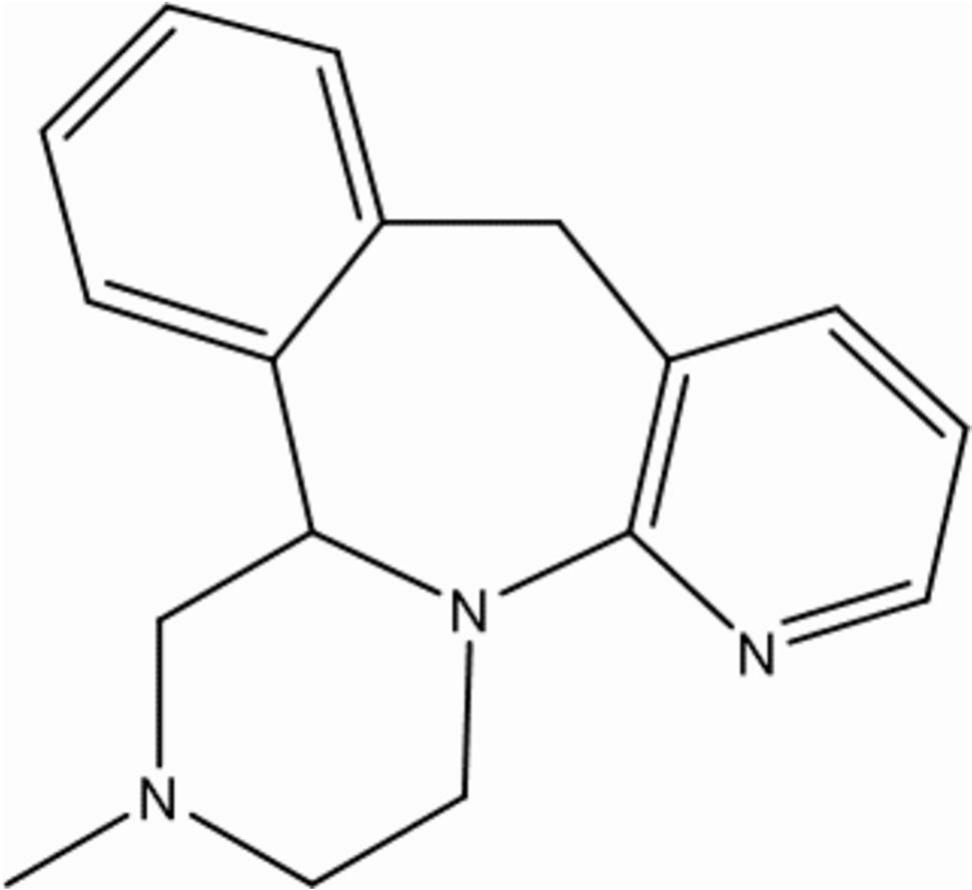
Chemical structure of MTZ

Mirtazapine (MTZ) is 5-methyl-2,5,19-triazatetracyclo [13.4.0.02,7.08,13] nonadeca-1(15),8,10,12,16,18-hexaene (Fig. 1), a noradrenergic and specific serotonergic antidepressant [1]. The empirical formula of MTZ is C17H19N3, and its molecular mass is 265.36 (Benjamin and Doraiswamy 2011). The α-2 antagonistic properties of the R(-)- and S( +)-enantiomers make them both pharmacologically active and may be a factor in their antidepressant effects, which are combined in the pill as a racemic combination (Delbressine et al. 1998). It was examined for neuropharmacological action under the name ORG-3770 by De Boer et al. (1988).

#### Pharmacokinetics

##### Absorption

MTZ is effectively absorbed from the gastrointestinal tract (Voortman and Paanakker 1995). The pharmacokinetics of MTZ are age- and sex-dependent. Adults and the elderly attain a steady state after 4 days and 6 days, respectively, after a once-daily oral dose (Voortman and Paanakker 1995; Timmer et al. 1996).

##### Distribution

Over a concentration range of 0.01 to 10 mcg/ml, 85% of MTZ is bound to plasma proteins. It was reported that MTZ binds to human plasma proteins in vitro at concentrations between 0.01 and 10 mg/L with an approximate 85% affinity (Van der Vorstenbosch and Delbressine 1988). Over the examined concentration range, binding was nonspecific and reversible. MTZ binds to human erythrocytes in vitro with a 40% affinity, comparable to the hematocrit of whole blood (Timmer et al. 2000).

##### Elimination

The liver is responsible for the majority of the biotransformation of MTZ. The main mechanisms include glucuronide conjugation, demethylation, and hydroxylation (Sandker et al. 1994). MTZ is considerably eliminated in the urine and feces after being digested. More than 75% of the prescribed dose is eliminated in the urine, leaving the remaining 20% in the feces. The amount of the dose that was excreted unchanged was less than 4% (Delbressine et al. 1998). The cytochrome P450 enzyme system is primarily involved in the oxidative pathways where MTZ is biotransformed (Dahl et al. 1997).

#### Pharmacodynamic of MTZ

##### Antidepressant

**Fig. 2:**
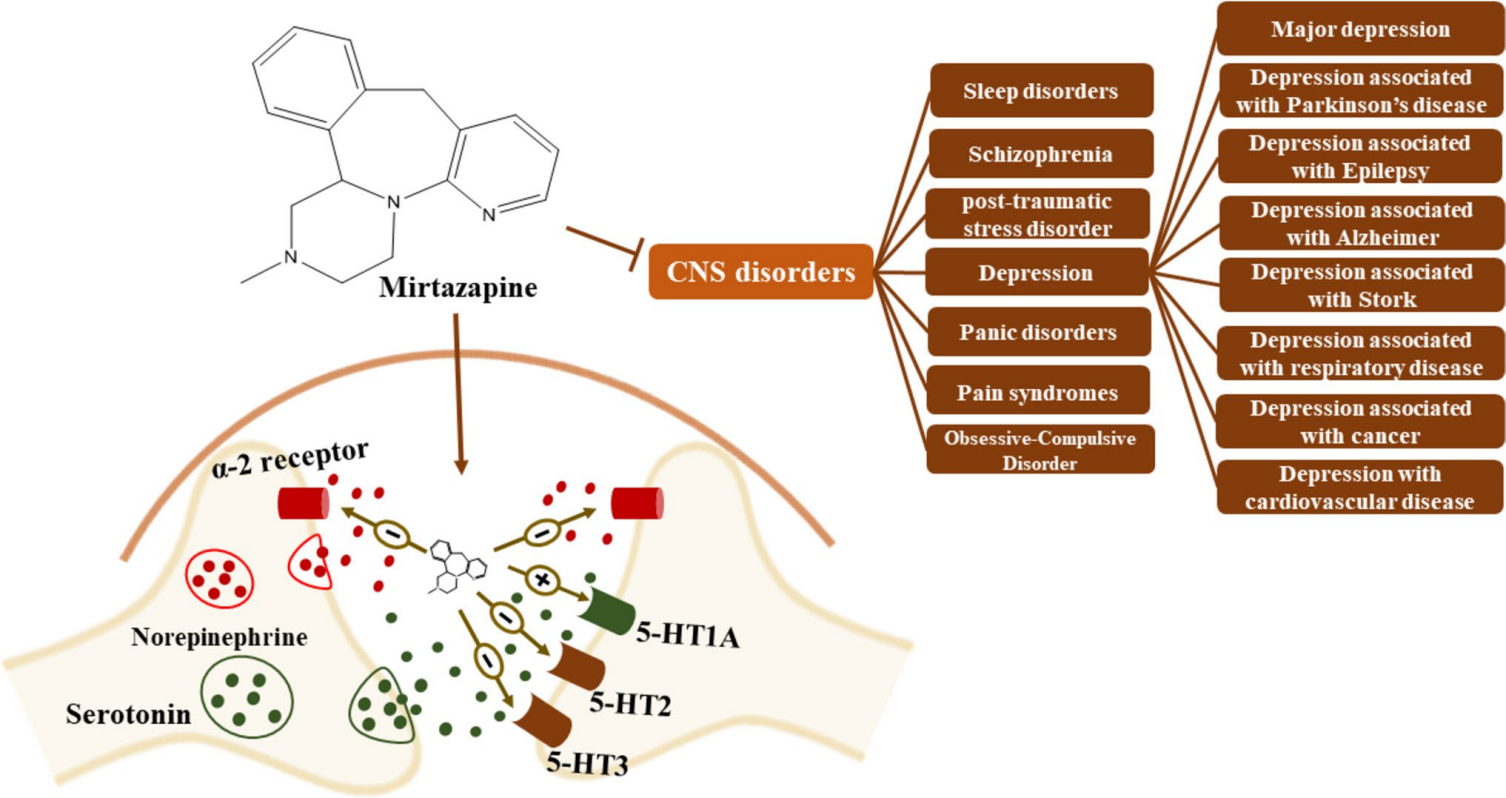
Effectiveness of MTZ in treating a major depressive disorder and depression associated with several conditions

MTZ is hypothesized to have antidepressant effects because of the synergy between noradrenergic and serotonergic actions. As a racemate, MTZ has pharmacological activity in both the S-( +)- and R-( −)-enantiomers [5]. By antagonism of central α_2_-adrenergic heteroreceptors and autoreceptors as well as postsynaptic inhibition of 5-HT2 and 5-HT3 receptors, MTZ increases noradrenergic and 5-HT1A-mediated serotonergic neurotransmission (Fig. 2) (De Boer et al. 1995). When compared to various other types of antidepressants, MTZ differs in that it does not impede the reuptake of serotonin or noradrenaline or monoamine oxidase activity (Pinder 1997b). Theoretically, MTZ’s blocking of the 5-HT2 and 5-HT3 receptors could lessen the risk of some serotonergic side effects linked to the nonselective activation of serotonin receptors by SSRIs (Pinder 1997a, 1997b).

MTZ has numerous additional pharmacodynamic effects in addition to its antidepressant action. Blocking 5-HT2 receptors, for instance, has been linked to better sleep (Pinder 1997b). Moreover, MTZ enhances the quality and duration of sleep and, in contrast to SSRIs, does not inhibit rapid eye movement sleep (Aslan et al. 2002; Winokur et al. 2003; Shen et al. 2006).

It is interesting to note that reuptake inhibitors can raise cortisol levels (Schule et al. 2003), and cortisol secretion is reduced by MTZ (Laakmann et al. 1999, 2004; Schüle et al. 2002; Schule et al. 2003). MTZ increases serotonergic transmission in a way that suggests that the medication may start working more quickly than selective serotonin reuptake inhibitors (SSRIs). Animal model studies, unlike SSRIs, have demonstrated that MTZ treatment increases the rate at which serotonin neurons fire (Holm and Markham 1999).

##### Antioxidant effect

The imbalance between the generation of free radicals and their neutralization by antioxidants is known as oxidative stress (OS). Increased ROS production and weaker antioxidant defenses lead to this redox imbalance. High levels of ROS can result in OS and cell death by causing inflammation and damage to macromolecules. As a result, OS is connected to the etiology of a variety of diseases (Rani et al. 2016; Mahmoud et al. 2017; Liguori et al. 2018). Numerous in vitro and in vivo investigations have clearly demonstrated the antioxidant and anti-inflammatory properties of MTZ (Elsisi et al. 2021; Hafez et al. 2021b). MTZ’s antioxidant function had a beneficial effect on many organs. MTZ increased the nonenzymatic antioxidant glutathione (GSH) and decreased lipid peroxidation in reproductive toxicity (El-Sisi et al. 2017). Additionally, other models of the antioxidant effect also accounted for renal ischaemia‒reperfusion injury (Tok et al. 2012) and peptic ulcers (Bilici et al. 2009). Tok et al. investigated MTZ’s impact on the OS induced by ischaemia‒reperfusion in rat kidneys. According to the findings, MTZ decreased renal MDA content and MPO enzymatic activity and GSH and glutathione-S-transferase (GST) activities. Moreover, the histopathological findings were attenuated by MTZ. Therefore, MTZ may protect against IR-induced kidney injury (Tok et al. 2012). Another study investigated the possible chemoprotective properties of MTZ against OS and DNA damage induced by cisplatin. Because of its antioxidant properties, MTZ exerts chemoprotective effects on DNA damage and OS induced by cisplatin in the rat brain, as proven by decreased MDA, MPO, and 8-OH-GUA levels and increased GSH levels (Gulec et al. 2013). Additionally, in BV2 microglia exposed to isoflurane, MTZ decreased the expression of the protein Iba1 and decreased the production of the proinflammatory mediators interleukin (IL)-1β and IL-18, which isoflurane produces by blocking the activation of the nod-like receptor family protein 3 (NLRP3) inflammasome in BV2 microglia. MTZ downregulates NADPH oxidase 4 (NOX4) expression and the production of reactive ROS. These findings led us to conclude that MTZ may be a possible intervention to stop cognitive damage induced by isoflurane exposure in clinical settings (Wang et al. 2022).

##### Anti-inflammatory effect

**Fig. 3:**
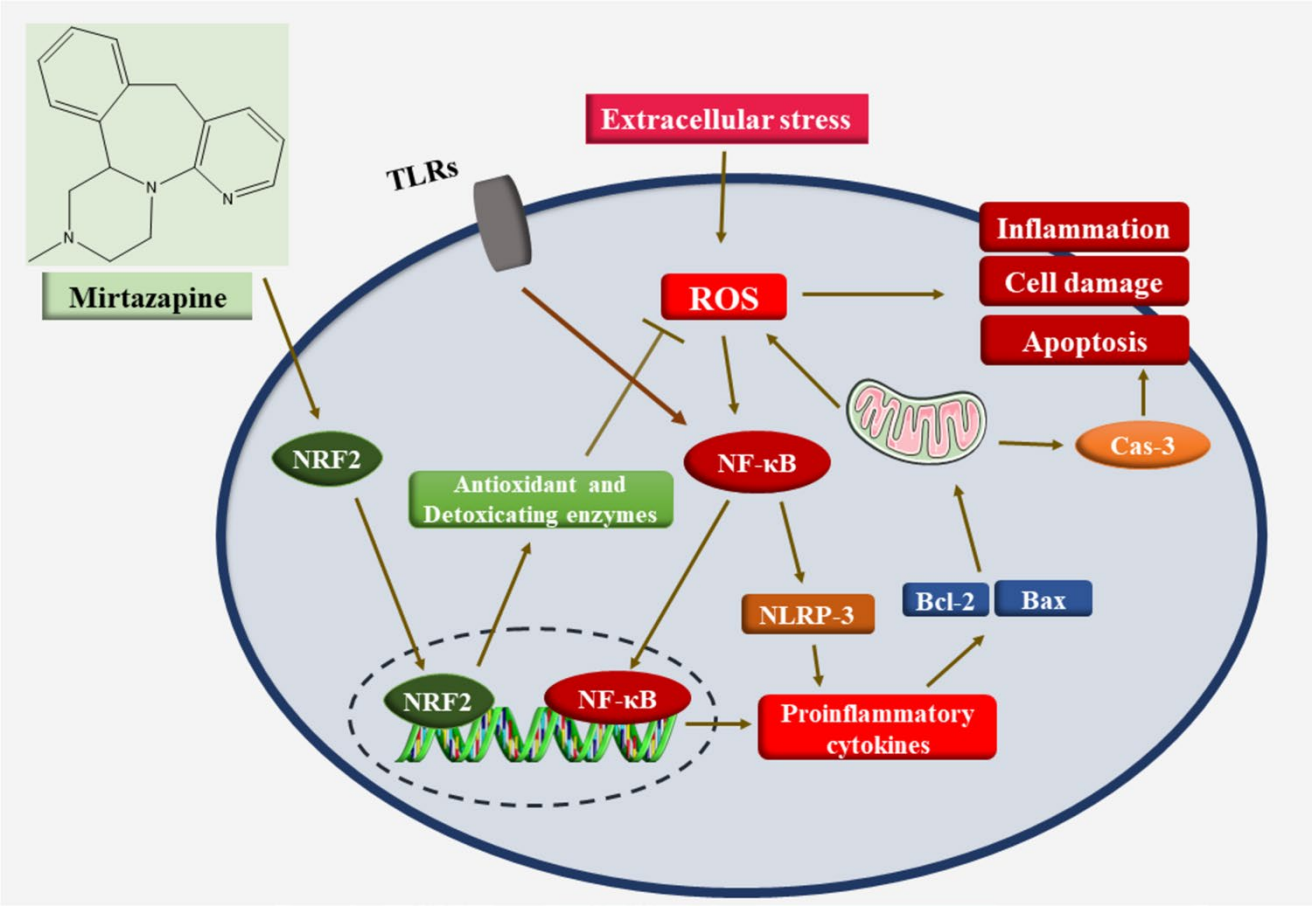
Molecular mechanisms underlying the antioxidant, anti-inflammatory, and anti-apoptotic activities of MTZ

Previous research has suggested that MTZ treatment for major depressive disorders has anti-inflammatory effects and decreases tumor necrosis factor (TNF)-α levels (Gupta et al. 2016). Similarly, in a model of neuropathic pain, MTZ reduced TNF-α and nuclear factor-kappa B (NF-κB) (Zhu et al. 2008), as well as in a model of immune-mediated liver damage (Almishri et al. 2019). Inhibiting the NLRP3/caspase-1/IL-18 signal has demonstrated mechanistically anti-inflammatory effects (Hafez et al. 2021a) (Fig. 3).

##### Anti-apoptotic effect

Mitochondrial membranes may be damaged by increased ROS generation, which will cause the release of cytochrome c (Maneesh and Jayalekshmi 2006). This, in turn, interacts with apoptosis protease activation factor-1 to activate caspase-9 as the initiator, which then activates caspase-3 to cause apoptosis (Sinha Hikim et al. 2003). El-Sisi et al. reported that MTZ can be used as a preventative medicine to stop the reduction in sperm count and viability caused by nitrofurazone. Additionally, both medications reduced the effects of nitrofurazone on rat testes MDA, GSH depletion, elevation of TNF-α, and decrease in c-kit levels. In terms of apoptosis, immunohistochemistry research showed that nitrofurazone-induced testicular tissue expressed caspase-3, which was decreased by chrysin and MTZ. Histopathological data further substantiated the preventive benefits of both medications. The findings revealed that OS and apoptotic tissue damage were suppressed by the cytoprotective effects of chrysin and MTZ on rat testes (El-Sisi et al. 2017). Consequently, chrysin and MTZ on rat testes are mediated by mitigating testicular oxidative injury and apoptosis. Additionally, Engel et al. showed that by lowering the expression of antiapoptotic proteins, MTZ decreased neuronal death in the hippocampus and cerebral cortex of mice by downregulating Bcl-xL, Bax, Bad, and p53 (Engel et al. 2013). Lieberknecht et al. reported that MTZ protects the brain against H2O2-induced cell death. In particular, MTZ decreased p53 mRNA expression and cell death (Lieberknecht et al. 2020).

## Pharmacological update of MTZ

In our review, we used the search engines of PubMed as well as databases of Scopus, Web of Science, and Google Scholar. The pharmacological update is summarized in Table 1 and discussed as follows:

**Table 1:** Summary of the pharmacological effects of MTZ

System or organ	Disease	Main effects	Reference
CNS	Major depression	Randomized, controlled trials that compared it to placebo and other antidepressants, such as fluoxetine, paroxetine citalopram, trazodone, amitriptyline, doxepin, clomipramine, imipramine, and venlafaxine	Richou et al. (1995), Bruijn et al. (1996), Wheatley et al. (1998), Leinonen et al. (1999), Guelfi et al. (2001), Benkert et al. (2002)
	Depression associated with Parkinson’s disease	MTZ affected PDPsy and effectively reduced hallucinations in PD	Sid-Otmane et al. (2020)
		MTZ effectively treated depression in PD patients as well as insomnia and psychosis	Pena et al. (2018)
	Depression associated with epilepsy	MTZ is generally safe and useful for patients with epilepsy	Górska et al. (2018)
	Depression associated with Alzheimer’s disease	MTZ produced a quick and long-lasting response in three individuals with AD and depression worsened by anxiety, sleeplessness, and weight loss The high frequency of these bothersome symptoms in depressed AD patients highlights the need for an antidepressant that combines beneficial effects on these symptoms and a good safety profile MTZ may play a role in treating depression that coexists with dementia, anxiety, sleeplessness, and weight loss, based on the clinical reactions of the three patients	Lyketsos and Olin (2002), Caraci et al. (2010)
		MTZ exhibited promising effects for AD patients who also have sadness, sleeplessness, anxiety, and weight loss	Raji and Brady (2001)
	Depression associated with stroke	In patients with an acute stroke, MTZ dramatically decreased the incidence of poststroke depression MTZ successfully treated poststroke depression	Niedermaier et al. (2004)
	Depression associated with respiratory diseases	MTZ has effects that are possibly helpful for breathlessness and elevates the mood, mostly by altering the processing and perception of afferent information in the brain MTZ’s ability to inhibit 5-HT2 and 5-HT3 receptors prevents it from sharing some of the negative effects of other regularly prescribed antidepressants, and inhibiting H1 receptors helps improve appetite and sleep for patients	Yang et al. (2020)
	Depression associated with cancer	In nausea and sleep disturbance, which are frequent and upsetting side effects of cancer, MTZ significantly reduced sadness, nausea, sleep disruption, pain, and quality of life in cancer patients	Kim et al. (2008)
		In a pilot open-label, in advanced cancer patients with pain and other disturbing symptoms, MTZ may be useful for treating numerous symptoms, depression, and quality of life in advanced cancer patients	Theobald et al. (2002)
		MTZ demonstrated benefits for early, medium, and late insomnia. Research shows that MTZ can help cancer patients with insomnia, anxiety, and depressive symptoms	Cankurtaran et al. (2008)
	Depression associated with cardiovascular disease	Within the MIND-IT study, a randomized controlled trial, MTZ is an effective and safe for MI who also had depression	Honig et al. (2007)
		A short retrospective cohort study concluded the effectiveness of MTZ for hospitalized individuals who were given MTZ following a mental consultation	Allen et al. (2020)
	Sleep disorders	In patients with major depressive illness who had poor sleep quality at weeks 1 and 2 of treatment, MTZ dramatically improved sleep continuity	Winokur et al. (2000)
		MTZ has a significant impact on slow-wave sleep When compared to a placebo, MTZ improved the factors connected to uninterrupted sleep. It reduced the quantity and length of awakenings while raising the sleep efficiency index Regarding characteristics related to rapid eye movement sleep, there was no discernible impact	Aslan et al. (2002)
		In alcohol abuse and social anxiety disorder, a total of 41.7% of them were categorized as respondents at the study’s conclusion. People with alcohol addiction frequently exhibit symptoms of social anxiety. On the other hand, MTZ reduced the social anxiety symptoms	Liappas et al. (2003)
		For 4–5 weeks after detoxification, a total of 54 inpatients with alcoholism were randomly assigned to undergo MTZ with psychotherapy or cognitive-behavioral treatment. The combination group significantly outperformed the control group in terms of statistically reducing social anxiety symptoms In a subsequent trial, MTZ was discovered to be beneficial during the detoxification of patients who were addicted to alcohol as opposed to venlafaxine	Liappas et al. (2005)
	Schizophrenia	Adding MTZ to haloperidol led to a statistically significant reduction in negative symptoms after 6 weeks of treatment in a double-blind, randomized, placebo-controlled study including 30 patients	Berk et al. (2001)
		Combination of MTZ with clozapine in an 8-week, double-blind, placebo-controlled experiment for the treatment of negative symptoms of schizophrenia. The results revealed that the treatment considerably reduced SANS scores compared to the placebo, especially on the avolition/apathy and anhedonia/asociality SANS subscales	Zoccali et al. (2004)
		MTZ treated akathisia caused by neuroleptics in twenty-six schizophrenia patients were randomized to receive either MTZ 15 mg or a placebo for 5 days in a double-blind study. The MTZ group displayed noticeably higher response rates	Poyurovsky et al. (2003)
	Posttraumatic stress disorder	Twenty-nine PTSD patients participated in this trial and were given MTZ or a placebo for 8 weeks. After 8 weeks of therapy, patients receiving MTZ had a response rate that was noticeably higher than those receiving a placebo, as determined. After 8 weeks of MTZ therapy, three open-label studies also discovered significant improvement rates	Connor et al. (1999), Bahk et al. (2002), Chung et al. (2004)
	Panic disorder	MTZ demonstrated encouraging effects in lowering depression and anxiety symptoms in panic disorders according to numerous open-label studies	Boshuisen et al. (2001), Carli et al. (2002), Sarchiapone et al. (2003)
	Pain syndromes	In several clinical studies, MTZ may be helpful for pain syndromes	Brannon and Stone (1999), Nutt and Law (1999), Brannon et al. (2000), Bendtsen and Jensen (2004)
		Twenty-four patients with persistent tension-type headaches In a randomized, double-blind, placebo-controlled crossover trial. After 8 weeks of treatment, MTZ significantly outperformed a placebo in the preventive treatment of persistent tension-type headaches	Bendtsen and Jensen (2004)
	Obsessive–compulsive disorder	The effectiveness of MTZ in patients with OCD has been examined in two randomized, controlled trials. MTZ’s effects on OCD have been documented to be effective in OCD as a pharmacotherapy	Koran et al. (2005)
		In a pilot trial, the effects of citalopram and MTZ in patients with OCD who did not also have comorbid depression When MTZ was combined with citalopram, they discovered a faster onset of responsive action in OCD symptoms and decreased undesirable side effects	Pallanti et al. (2004)
The potential protective effects of MTZ on diseases other than CNS-related disorders			
Liver diseases	High-fat diet mice model	Obese mice treated with MTZ had reduced levels of body weight, serum TG and AST levels The treated mice had lower blood glucose levels and increased insulin sensitivity and glucose transporter 4 expression levels	Wu et al. (2020)
	Immune-mediated liver disease	MTZ rapidly shifts the hepatic B-cell populations and functional cytokine signatures of mice Treatment with MTZ increases the retention of innate-like B cells that express CXCR3, producing more anti-inflammatory cytokines	Almishri et al. (2021)
	IV infection with a pathogenic strain of *S. aureus* in mice	MTZ affects liver innate immunity and inhibits immune-driven activation of hepatic macrophages Treatment with MTZ may have beneficial effects on sepsis, and it significantly decreases the risk of hepatic abscess formation	Davis et al. (2020)
	Liver fibrosis induced by thioacetamide in mice	MTZ treatment ameliorated TAA-induced liver fibrosis by lowering portal BP, liver procollagen I content, and α-SMA expression MTZ-treated animals had significantly less hepatic collagen accumulation. MTZ decreased the levels of protein kinase C, TGF-β1, phosphorylated Smad3, p-ERK1/2 MTZ significantly reduced oxidative stress, by declining hepatic lipid peroxidation and NADPH oxidase 1 and increased GSH content	El-Tanbouly et al. (2017)
Kidney diseases	Diabetic nephropathy in rats	MTZ downregulated NLRP3 and caspase-1 expression as well as the renal level of IL-1β in diabetic rats MTZ may be effective in the management of DM and other metabolic illnesses	Sahin et al. (2019)
Cardiovascular diseases related to CNS disorders	An in vitro human plasma-based study	The antiplatelet effects MTZ are mediated by 5-HT2A and α2-adrenergic receptors blocking	Kawano et al. (2022)
Diabetes	Type 1 diabetic rats	In T1DM rat models, MTZ reduced GLUT2 by changing the expression of leptin and galanin in the livers of type 1 diabetic rats with hyperglycaemia. These findings concluded that patients with T1DM can use MTZ as an antidepressant and as a medication to lower blood glucose levels	Bektur et al. (2019a)
	Diabetes-induced hyperalgesia in rats	MTZ exhibits beneficial effects in diabetes-induced hyperalgesia is mediated by decreasing TRPV1 and ASIC1 levels	Bektur et al. (2019b)
GIT	Nausea and vomiting in chemotherapy-treated cancer patients	In a study of 35 patients, when given in conjunction with platinum-based regimens to patients with thoracic cancer, adding MTZ to the recommended antiemetic regimen for CINV may be advantageous with appropriate tolerability	Kinomura et al. (2023)
Heavy metal-induced neurotoxicity	Cd-induced neurotoxicity in rats	MTZ attenuates Cd-induced neurotoxicity by upregulating the transcription factor Nrf2, suppressing NF-κB/TLR4 signalling, MTZ effectively decreasing TNF-α, IL-1β, and IL-6 MTZ reduced histological abrasions in the dentate gyrus, CA1 and CA3 regions of the hippocampus, and cerebral cortex of the rat brain	Alharthy et al. (2023)
Reproductive diseases	CP-induced oxidative stress in rat ovaries	CP-induced oxidative stress in rat ovaries causes infertility in female rats. MTZ could counteract this impact and safeguard fertility against CP-induced damage	Khedr (2015)
	Cisplatin-induced infertility in rats	MTZ decreased the levels of MDA, MPO, NO, and 8-hydroxy-2 deoxyguanine and effectively increased GSH, GPx, and SOD levels	Altuner et al. (2013)
	Nitrofurazone-induced testicular injury in rats	MTZ stopped the reduction in sperm count and viability caused by nitrofurazone MTZ reduced the effects of nitrofurazone on rat testes MDA, GSH depletion, elevation of TNF-α, and decrease in c-kit levels MTZ attenuated apoptosis by downregulating testicular caspase-3	El-Sisi et al. (2017)
	Four case reports of hot flushes and sweating episodes in women	MTZ exhibited promising effects on the frequency and intensity of hot flushes and sweating episodes in women, MTZ appears to significantly lessen both of these symptoms	Waldinger et al. (2000)
Pruritus	Patients with chronic pruritus: a pilot study	MTZ has shown success in lowering itch severity is MTZ, a dual noradrenergic and serotonergic antidepressant with antihistaminic characteristics	Hundley and Yosipovitch (2004), Lee et al. (2016)

### CNS disorders

#### Major depression

MTZ is effective in treating a major depressive disorder and in depression associated with several conditions (Fig. 2**)** in several randomized, controlled trials that compared it to placebo and other antidepressants, such as fluoxetine, paroxetine citalopram, trazodone, amitriptyline, doxepin, clomipramine, imipramine, and venlafaxine (Richou et al. 1995, Bruijn et al. 1996, Wheatley et al. 1998, Leinonen et al. 1999, Guelfi et al. 2001, Benkert et al. 2002).

#### Depression associated with Parkinson’s disease

Parkinson’s disease (PD) patients frequently experience psychotic symptoms, and a set of criteria for PD with psychosis (PDPsy) has been defined. Hallucinations are one of the specific symptoms that fit this description, with visual hallucinations being the most frequent. According to research by Tagai et al., the use of MTZ in a patient with PDPsy reduced the patient’s refractory psychotic symptoms, particularly visual hallucinations, without worsening motor symptoms (Tagai et al. 2013). The effects of MTZ and other antidepressants on mild depression linked to PD were also documented by Costa et al. (2012). Sid-Otmane et al. investigated how MTZ and other antidepressants affected PDPsy and found that they effectively reduced hallucinations in PD (Sid-Otmane et al. 2020). Antidepressant medications, such as MTZ, are effective in treating depression in PD patients as well as insomnia and psychosis, according to Pena et al. (2018).

#### Depression associated with epilepsy

Mood issues are frequently co-occurring in people with epilepsy. The lifetime prevalence ranges from 11 to 62% (Stanisław Wiglusz et al. 2012). Depression is one of the main psychiatric conditions that has a detrimental effect on the quality of life of people with epilepsy (Mula 2017). Epilepsy and depression may be related in a bidirectional manner; having epilepsy would seem to increase the risk of depression while having depression would seem to increase the chance of epilepsy (Forsgren and Nyström, 1990). Epilepsy patients who are depressed attempt suicide four to five times more frequently than people without epilepsy (Matthews and Barabas 1981; Batzel and Dodrill 1986; Blumer 2002). According to clinical studies by Natalia et al., antidepressants, including MTZ, are generally safe for patients with epilepsy when used at therapeutic dosages (Górska et al. 2018).

#### Depression associated with Alzheimer’s disease

Up to 10% of Americans over the age of 65 in the USA have Alzheimer’s disease (AD), which is the most frequent cause of dementia. The main factor in the early hospitalization of these patients is frequent psychiatric and behavioral difficulties. A total of 10–30% of AD patients meet the diagnostic requirements for severe depression (Mann et al. 1989). MTZ produced a quick and long-lasting response in three individuals with AD and depression worsened by anxiety, sleeplessness, and weight loss. The high frequency of these bothersome symptoms in depressed AD patients highlights the need for an antidepressant that combines beneficial effects on these symptoms and a good safety profile. MTZ may play a role in treating depression that coexists with dementia, anxiety, sleeplessness, and weight loss, based on the clinical reactions of the three patients (Lyketsos and Olin 2002; Caraci et al. 2010). Because brain serotonergic and noradrenergic neurotransmission regulate mood, sleep, and hunger, MTZ may be beneficial in treating depressed AD patients. The selection of an antidepressant should be based on a patient’s unique medical and psychological profile due to the absence of comparative data. MTZ exhibited promising effects for AD patients who also have sadness, sleeplessness, anxiety, and weight loss (Raji and Brady 2001).

#### Depression associated with stroke

The most prevalent and demanding neuropsychiatric poststroke consequences are those involving depression, which can occur after a stroke (Shi et al. 2017; Taylor-Rowan et al. 2019). One of the most frequent aftereffects of a stroke is poststroke depression, which affects 20 to 40% of all patients. In patients with an acute stroke, MTZ dramatically decreased the incidence of poststroke depression. Additionally, the study showed that this antidepressant successfully treated poststroke depression. Only 5.7% (2/35) of the patients receiving MTZ treatment and 40% (14/35) of the untreated patients experienced poststroke depression. Sixteen patients in total had poststroke depression; however, 15 of them recovered after beginning MTZ therapy (Niedermaier et al. 2004).

#### Depression associated with respiratory diseases

Most chronic obstructive pulmonary and interstitial lung disease patients experience breathlessness as a frequent and bothersome symptom of advanced disease (Solano et al. 2006; Moens et al. 2014). MTZ has effects that are possibly helpful for breathlessness and elevates the mood, mostly by altering the processing and perception of afferent information in the brain MTZ’s ability to inhibit 5-HT2 and 5-HT3 receptors prevents it from sharing some of the negative effects of other regularly prescribed antidepressants, and inhibiting H1 receptors helps improve appetite and sleep for patients (Yang et al. 2020).

#### Depression associated with cancer

Kim et al. assessed the effectiveness of oral disintegrating tablets of MTZ for nausea and sleep disturbance, which are frequent and upsetting side effects of cancer. MTZ significantly reduced sadness, nausea, sleep disruption, pain, and quality of life in cancer patients (Kim et al. 2008). MTZ was tested in a pilot open-label, crossover study by Theobald et al. in advanced cancer patients with pain and other disturbing symptoms. They examined how MTZ therapy affected patients’ levels of depressive symptoms, the severity of their pain, appetites, sleep patterns, weight, and general quality of life. According to this open-label pilot trial, MTZ may be useful for treating numerous symptoms, depression, and quality of life in advanced cancer patients (Theobald et al. 2002). Cankurtaran et al. investigated the efficacy of MTZ and imipramine on depression and anxiety symptoms as well as pain, nausea, vomiting, appetite loss, and sleep disruptions. Pain, nausea, vomiting, and appetite loss did not differ significantly between the three visits in the MTZ, imipramine, or control groups. Only the MTZ group demonstrated benefits for early, medium, and late insomnia. Research shows that MTZ can help cancer patients with insomnia, anxiety, and depressive symptoms (Cankurtaran et al. 2008).

#### Depression associated with cardiovascular disease

Studies examining the cardiovascular effects of MTZ in various at-risk populations are available. Within the MIND-IT study, a nested randomized controlled trial examined the effectiveness and safety of MTZ administered for 24 weeks to 331 adults hospitalized with an MI who also had depression. In comparison to the placebo, MTZ did not affect blood pressure, heart rate, QT, or QRS interval (Honig et al. 2007). These results imply that, at least temporarily, MTZ may be safe for individuals who have recently experienced a cardiac episode with a history of CVD. A short retrospective cohort study examined 61 medically hospitalized individuals who were given MTZ following a mental consultation (Allen et al. 2020).

### CNS disorders other than depression

#### Sleep disorders

The majority of people with severe depressive illness subjectively state interrupted sleep or prolonged sleep onset (Winokur and Reynolds 1994). In patients with major depressive illness who had poor sleep quality at weeks 1 and 2 of treatment, Winokur et al. found that MTZ dramatically improved sleep continuity. During the first week, MTZ considerably reduced sleep latency and significantly enhanced total sleep time and sleep efficiency. Similar results were seen following the second week. MTZ did not significantly alter rapid eye movement sleep characteristics (Winokur et al. 2000). Aslan et al. examined how healthy volunteers’ sleep was affected by a single dose of MTZ. The results indicate that the MTZ has a significant impact on slow-wave sleep. It is advised that more research be done on the effectiveness of antidepressants concerning how 5-HT2 blockage affects slow-wave sleep. When compared to a placebo, MTZ improved the factors connected to uninterrupted sleep. It reduced the quantity and length of awakenings while raising the sleep efficiency index. Regarding characteristics related to rapid eye movement sleep, there was no discernible impact (Aslan et al. 2002).

In alcohol abuse and social anxiety disorder, a total of 41.7% of them were categorized as respondents at the study’s conclusion. People with alcohol addiction frequently exhibit symptoms of social anxiety. On the other hand, MTZ reduced the social anxiety symptoms (Liappas et al. 2003). For 4–5 weeks after detoxification, a total of 54 inpatients with alcoholism were randomly assigned to undergo MTZ with psychotherapy or cognitive-behavioral treatment. The combination group significantly outperformed the control group in terms of statistically reducing social anxiety symptoms. In a subsequent trial, MTZ was discovered to be beneficial during the detoxification of patients who were addicted to alcohol as opposed to venlafaxine (Liappas et al. 2005).

#### Schizophrenia

Schizophrenia is a severe mental condition that can be fatal for both the sufferers and those who care for them. Anhedonia, avolition, social disengagement, poverty of thinking, and cognitive dysfunction are some of the negative symptoms of schizophrenia. Delusions, hallucinations, and thought disorders are forms of positive symptoms (Saha et al. 2005; Salavati et al. 2015; Yang and Tsai 2017). MTZ was evaluated in two randomized, double-blind, placebo-controlled trials for the management of the negative symptoms of schizophrenia (Berk et al. 2001; Zoccali et al. 2004). In both studies, MTZ was added to an antipsychotic as a supplement. Berk et al. observed that adding MTZ to haloperidol led to a statistically significant reduction in negative symptoms after 6 weeks of treatment in a double-blind, randomized, placebo-controlled study including 30 patients (Berk et al. 2001). For the treatment of negative symptoms of schizophrenia, MTZ was combined with clozapine in an 8-week, double-blind, placebo-controlled experiment. The results revealed that the treatment considerably reduced SANS scores compared to the placebo, especially on the avolition/apathy and anhedonia/asociality SANS subscales (Zoccali et al. 2004). In another study, the efficacy of MTZ in treating akathisia caused by neuroleptics was investigated. Twenty-six schizophrenia patients were randomized to receive either MTZ 15 mg or a placebo for 5 days in a double-blind study. The MTZ group displayed noticeably higher response rates (Poyurovsky et al. 2003).

#### Posttraumatic stress disorder

The effectiveness of MTZ in treating posttraumatic stress disorder (PTSD) is being studied in several trials, one of which is a randomized, double-blind, placebo-controlled experiment (Davidson et al. 2003). Twenty-nine PTSD patients participated in this trial and were given MTZ or a placebo for 8 weeks. After 8 weeks of therapy, patients receiving MTZ had a response rate that was noticeably higher than those receiving a placebo, as determined. After 8 weeks of MTZ therapy, three open-label studies also discovered significant improvement rates (Connor et al. 1999; Bahk et al. 2002; Chung et al. 2004).

#### Panic disorder

One randomized double-blind experiment examining MTZ’s effectiveness for treating panic disorder has been published thus far (Ribeiro et al. 2001). Twenty-seven outpatients were randomized to an 8-week therapy of either fluoxetine or MTZ in a flexible-dose design following a 1-week placebo run-in. At the study’s conclusion, patients from both groups demonstrated statistically significant improvement; however, no differences between the two therapy groups could be seen. MTZ demonstrated encouraging effects in lowering depression and anxiety symptoms in panic disorders according to numerous open-label studies (Boshuisen et al. 2001; Carli et al. 2002; Sarchiapone et al. 2003).

#### Pain syndromes

In several clinical studies, MTZ may be helpful for pain syndromes (Brannon and Stone 1999; Nutt and Law 1999; Brannon et al. 2000; Bendtsen and Jensen 2004). Twenty-four patients with persistent tension-type headaches were enrolled in the Bendtsen et al. study, which was a randomized, double-blind, placebo-controlled crossover trial. After 8 weeks of treatment, MTZ significantly outperformed a placebo in the preventive treatment of persistent tension-type headaches (Bendtsen and Jensen 2004). The results from an open-label study also point to MTZ as a potential fibromyalgia treatment (Samborski et al. 2004).

#### Obsessive–compulsive disorder

Many individuals with obsessive–compulsive disorder (OCD) show little improvement with serotonin reuptake medications, which are the standard of care. Through a mechanism different from reuptake inhibition, MTZ improves serotonergic activity. The effectiveness of MTZ in patients with OCD has been examined in two randomized, controlled trials. MTZ’s effects on OCD have been documented to be effective in OCD as a pharmacotherapy (Koran et al. 2005). In a pilot trial, Pallanti et al. investigated the effects of citalopram and MTZ in patients with OCD who did not also have comorbid depression. When MTZ was combined with citalopram, they discovered a faster onset of responsive action in OCD symptoms and decreased undesirable side effects. The citalopram plus MTZ group had effects earlier than the citalopram plus placebo group. Responders were higher in the citalopram + MTZ group in the fourth week of treatment (Pallanti et al. 2004).

## The potential protective effects of MTZ on diseases other than CNS-related disorders

### Liver diseases

In mice fed a high-fat diet, Cheng et al. inspected the effects of MTZ on metabolic parameters. The results showed that compared to untreated mice, obese mice treated with MTZ had reduced levels of body weight, serum TG, and AST levels. Additionally, the treated mice had lower blood glucose levels and increased insulin sensitivity and glucose transporter 4 expression levels. In conclusion, type 2 diabetes mellitus (DM) with hyperglycemia may be improved by MTZ treatment (Wu et al. 2020).

Treatment with MTZ quickly changes hepatic B cell populations, increasing the retention of innate-like B cells that express CXCR3 and produce a more anti-inflammatory effect. Hepatic B cell changes induced by MTZ may constitute a novel therapeutic strategy for treating immune-mediated liver disorders with B cell-driven pathology. According to research by Almishri et al., the antidepressant MTZ rapidly shifts the hepatic B cell populations and functional cytokine signatures of mice. Treatment with MTZ increases the retention of innate-like B cells that express CXCR3, producing more anti-inflammatory cytokines. B cell-driven pathology associated with immune-mediated liver illnesses may be treated uniquely by MTZ-induced hepatic B cell shifts (Almishri et al. 2021). According to Davis et al., MTZ also affects liver innate immunity and inhibits immune-driven activation of hepatic macrophages. Treatment with MTZ may have beneficial effects on sepsis, and it significantly decreases the risk of hepatic abscess formation (Davis et al. 2020).

The effects of MTZ were examined by El-Tanbouly et al. in a mouse model of liver fibrosis induced by thioacetamide. MTZ treatment ameliorated TAA-induced liver fibrosis by lowering portal BP, liver procollagen I content, and α-SMA expression. Additionally, as determined by Masson’s trichrome staining, MTZ-treated animals had significantly less hepatic collagen accumulation. MTZ decreased the levels of protein kinase C, transforming factor β1 (TGF-β1), phosphorylated Smad3 (p-Smad), and phosphorylated extracellular signal-regulated kinases 1 and 2 (p-ERK1/2). Furthermore, MTZ significantly reduced oxidative stress, as seen by declining hepatic lipid peroxidation and NADPH oxidase 1 and increased GSH content (El-Tanbouly et al. 2017).

### Kidney diseases

Sahin et al. investigated the antidepressant and antioxidant effects of MTZ in diabetic nephropathy. In this work, nod-like receptor family protein 3 NLRP3 and caspase-1 expression as well as the renal level of IL-1β were decreased in diabetic rats by MTZ administration. These findings imply that MTZ may be effective in the management of DM and other metabolic illnesses (Sahin et al. 2019).

### Cardiovascular diseases related to CNS disorders

According to Kawano et al., MTZ significantly suppressed both the synergistic interaction of serotonin (5-HT) and adrenaline as well as the synergistic interaction of ADP and either 5-HT or adrenaline. The antiplatelet effects MTZ are mediated by 5-HT2A and α2-adrenergic receptors blocking (Kawano et al. 2022).

### Diabetes

In T1DM rat models, MTZ reduced GLUT2 by changing the expression of leptin and galanin in the livers of type 1 diabetic rats with hyperglycemia. These findings concluded that patients with T1DM can use MTZ as an antidepressant and as a medication to lower blood glucose levels (Bektur et al. 2019a). Additionally, MTZ exhibits beneficial effects in diabetes-induced hyperalgesia. Since TRPV1 and ASIC1 levels are elevated in diabetic rats, MTZ’s suppressive influence on these levels may be one of the pharmacological mechanisms behind the drug’s therapeutic effects against diabetes-induced hyperalgesia (Bektur et al. 2019b).

### Nausea and vomiting in chemotherapy-treated cancer patients

In a study of 35 patients, when given in conjunction with platinum-based regimens to patients with thoracic cancer, adding MTZ to the recommended antiemetic regimen for CINV may be advantageous with appropriate tolerability (Kinomura et al. 2023).

### Heavy metal-induced neurotoxicity

A recent study examined the antioxidant and anti-inflammatory properties of MTZ against Cd-induced neurotoxicity. MTZ attenuates Cd-induced neurotoxicity by upregulating the transcription factor Nrf2, suppressing NF-κB/TLR4 signalling, and effectively decreasing TNF-α, IL-1β, and IL-6. Additionally, MTZ reduced histological abrasions in the dentate gyrus, CA1 and CA3 regions of the hippocampus, and cerebral cortex of the rat brain (Alharthy et al. 2023).

### Reproductive diseases

#### Infertility

Khedr studied MTZ and hesperidin’s protective effects against cyclophosphamide-induced infertility in rat ovaries. The findings imply that cyclophosphamide (CP)-induced oxidative stress in rat ovaries causes infertility in female rats. Hesperidin and MTZ could counteract this impact and safeguard fertility against CP-induced damage (Khedr 2015).

The effect of MTZ on cisplatin-induced infertility was explored by Altuner et al. MTZ decreased the levels of MDA, MPO, NO, and 8-hydroxy-2 deoxyguanine and effectively increased GSH, GPx, and SOD levels. In conclusion, cisplatin-induced oxidative stress in rat ovarian tissue results in infertility in female rats. This is reversed by MTZ in a dose-dependent manner (Altuner et al. 2013).

El-Sisi et al. examined whether chrysin and MTZ could protect rat testicles from nitrofurazone-induced injury. In this study, chrysin and MTZ were used as preventative medicines to reduce the increase in blood acid phosphatase enzyme activity and to stop the reduction in sperm count and viability caused by nitrofurazone. Additionally, both medications reduced the effects of nitrofurazone on rat testes MDA, GSH depletion, elevation of TNF-α, and decrease in c-kit levels. In terms of apoptosis, immunohistochemistry research showed that nitrofurazone-induced testicular tissue expressed caspase-3, which was decreased by chrysin and MTZ. Histopathological data further substantiated the preventive benefits of both medications (El-Sisi et al. 2017).

#### Hot flushes

Hot flushes are frequently associated with a drop in estrogen production, which is mostly brought on by menopause (Utian 1972). In the majority of patients, hormone replacement therapy (HRT) relieves 67% of women’s symptoms around menopause. HRT has been acknowledged as the first line of defense against menopausal hot flushes; however, it is contraindicated in a variety of clinical entities (Staropoli et al. 1998). According to Marcel et al. evaluations of the medication’s impact on the frequency and intensity of hot flushes and sweating episodes in women, MTZ appears to significantly lessen both of these symptoms (Waldinger et al. 2000).

### Pruritus

A frequent disease that can disrupt sleep and lower quality of life is chronic pruritus. The current approach to treating chronic itching targets the root of the problem, which may be dermatologic, systemic, or psychological in origin (Dhand and Aminoff 2014). Topical emollients, topical corticosteroids, and antihistamines are frequently the first treatments used in first-line therapy. For patients with persistent pruritus, GABA receptor modulators, opioid agonists/antagonists, and phototherapy can be employed (Dhand and Aminoff 2014). Recalcitrant itch is a bothersome symptom that requires a safe and efficient treatment (Davis et al. 2003; Kaur and Sinha 2018). Recent studies have shown that oral antidepressants are effective at reducing chronic itching caused by dermatologic, systemic, and psychogenic causes (Boozalis et al. 2018, 2019). One such antidepressant that has shown success in lowering itch severity is MTZ, a dual noradrenergic and serotonergic antidepressant with antihistaminic characteristics (Hundley and Yosipovitch 2004; Lee et al. 2016).

## Safety and toxicity of MTZ

MTZ has an excellent safety and tolerability profile. The overall incidence rate of adverse clinical events among patients receiving MTZ (65%) was lower than that of those receiving placebo (76%) or amitriptyline (87%) treatment (Anttila and Leinonen 2001).

Despite higher MTZ dosages, the majority of adverse effects were minor, temporary, and became progressively less intense and frequent with time. Low-dose-associated sleepiness and weight gain are often reported side effects of MTZ therapy, and they may be connected to the drug’s affinity for the antihistaminic (H_1_) receptor. In short-term trials, sedation or sleepiness was noted at low dosages and decreased in both intensity and frequency or progressively vanished during the titration of MTZ to higher levels (Bremner 1995; Anttila and Leinonen 2001). It is hypothesized that noradrenergic stimulation dominates the antihistaminic activity at larger doses (Barkin 1997; Nelson 1997; Fawcett and Barkin 1998), although these side effects occurred less frequently in European studies due, in part, to greater doses and were also shown to diminish over time; a much higher percentage of patients treated with low doses of MTZ experienced an increase in appetite and weight gain (Anttila and Leinonen 2001).

During the clinical development program, MTZ was often not linked to changes in laboratory parameters or cardiovascular vital signs. Some MTZ-treated patients showed a small increase in body weight. Most negative MTZ-related encounters occurred in the first few weeks of treatment (Fawcett and Barkin 1998). The psychomotor impairment caused by the initial dose of MTZ may have an impact on driving abilities; however, this does not last throughout treatment (Ramaekers et al. 1998; Ridout et al. 2003; Wingen et al. 2005).

## Conclusion and future recommendation

MTZ is an FDA-approved, effective, and well-tolerated drug for treating depression. MTZ is hypothesized to have antidepressant effects because of the synergy between noradrenergic and serotonergic actions and is effective in treating major depressive disorder and depression associated with epilepsy, Alzheimer’s disease, stroke, cardiovascular disease, and respiratory disease. Moreover, studies have reported the effectiveness of MTZ on other CNS disorders, such as schizophrenia, dysthymia, social anxiety disorder, alcohol dependency, posttraumatic stress disorder, panic disorder, pain syndromes, obsessive–compulsive disorder, and sleep disorders. Additionally, MTZ is potentially therapeutic against liver, kidney, cardiovascular, respiratory, infertility, heavy metal-induced neurotoxicity, and pruritus. In cancer patients, MTZ significantly reduced sadness, nausea, sleep disruption, and pain and improved quality of life. MTZ potentially protects against chemotherapy-induced toxicities such as cyclophosphamide-induced oxidative damage and infertility in rat ovaries. MTZ appears to significantly lessen the frequency and intensity of hot flushes and sweating episodes in women. These promising effects are mediated by potent antioxidant, anti-inflammatory, and anti-apoptotic effects. These positive outcomes of the scientific investigations motivate more and more clinical trials for a golden exceptional antidepressant.

## Data Availability

Data sharing not applicable to this article as no datasets were generated or analyzed during the current study.
